# An Insight on the Biomedical Potential of Portuguese Propolis from Gerês

**DOI:** 10.3390/foods11213431

**Published:** 2022-10-29

**Authors:** Carina Araújo, Rafaela Dias Oliveira, Filipa Pinto-Ribeiro, Cristina Almeida-Aguiar

**Affiliations:** 1 Biology Department, University of Minho, 4710-057 Braga, Portugal; 2 Life and Health Sciences Research Institute (ICVS), University of Minho, 4710-057 Braga, Portugal; 3 ICVS/3B’s—PT Government Associate Laboratory, 4710-057 Braga/4806-909 Guimarães, Portugal; 4 CBMA—Centre of Molecular and Environmental Biology, University of Minho, 4710-057 Braga, Portugal

**Keywords:** Portuguese propolis, osteoarthritis, chemical composition, antioxidant activity, anti-inflammatory activity

## Abstract

Osteoarthritis (OA), a progressive degenerative disease of weight-bearing joints, is the second leading cause of disability in the world. Despite all the advances and research over the last years, none of the proposed strategies has been effective in generating functional and long-lasting tissue. Due to the high prevalence of OA and the urgent need for an effective and successful treatment, interest in natural products as anti-inflammatory agents, such as propolis and its components, has emerged. In this work, we estimate the biomedical potential of Portuguese propolis, evaluating the in vitro antioxidant and anti-inflammatory effects of single hydroalcoholic extracts prepared with propolis from Gerês sampled over a five-year period (2011–2015) (G.EE_70_ and G.EE_35_). The in vivo and in vitro anti-inflammatory potential of the hydroalcoholic extract of mixtures of the same samples (mG.EE_70_ and mG.EE_35_) was evaluated for the first time too. DPPH• radical scavenging and superoxide anion scavenging assays showed the strong antioxidant potential of both hydroalcoholic extracts, either prepared from single propolis samples or from the mixtures of the same samples. Results also revealed an anti-inflammatory effect of mG.EE_35,_ both in vitro by inhibiting BSA denaturation and in vivo in the OA-induced model by improving mechanical hyperalgesia as well as the gait pattern parameters. Results further support the use of propolis blends as a better and more efficient approach to take full advantage of the bioactive potential of propolis.

## 1. Introduction

Osteoarthritis (OA) is a chronic progressive, degenerative, and multifactorial diarthrodial joint disease that mostly affects the hips, knees, hands, and feet [1,2,3]. This disorder is ranked as the second leading cause of disability in the world by the World Health Organization (WHO) and it causes a progressive loss and degradation of articular cartilage, development of osteophyte, subchondral bone sclerosis, and synovial inflammation [1,4]. OA incidence is rising at an alarming rate, with it being estimated that 35% of the world’s population will suffer from this condition by 2030 [5,6]. Taking this into consideration, OA is currently considered one of the major public health problems [7,8].

Osteoarthritis is classified as a low-grade inflammatory disease [4]. Although the pathology of OA remains controversial and unexplained, some factors, including aging, obesity, inflammation, trauma, joint overuse, metabolic disorders, genetic predisposition, and mechanical stress are considered influencing and risk variables [7,9,10], with aging being the most prominent of all of these factors [11]. Inflammatory factors, most notably interleukin-1ß, also play a critical role in OA pathological development [12,13].

Several therapeutic strategies have been developed and proposed to improve the restoration of the articular cartilage, but none has been effective in generating functional and long-lasting tissue [1,14]. Therefore, due to the high prevalence of OA and the urgent need for an effective treatment, the search for therapeutic alternatives, such as phytochemicals from plants and natural extracts, that can minimize side effects and be adapted to the progressive and multimodal character of OA has increased [1,15]. In fact, several natural compounds have shown anti-inflammatory potential, creating favorable conditions for the treatment development [16,17]. 

Propolis, or “bee glue”, is a brownish sticky resinous product that is produced by worker honeybees (mainly *Apis mellifera* L.) by mixing resin from plant exudates and buds with ß-glucosidase and other products of bees’ metabolism [18,19,20]. The chemical composition of this natural product is very complex and highly variable, depending on several macro and micro-geographical factors, such as the surrounding vegetation, geographic localization, climate, harvesting site and time, and bee species [18,20,21]. As a result, the lack of standardization coupled with this significant variability, is one of the main challenges associated with the use of propolis as a therapeutic agent [20,22]. Currently, more than 800 compounds have been identified in propolis samples [23], with flavonoids and phenolic compounds being the major propolis components responsible for its biological activities [24,25,26,27]. Multiple pharmacological properties have indeed been ascribed to propolis [20], including antioxidant [28,29] and anti-inflammatory capacities [30,31]. Propolis has been shown to offer exceptional protection for cartilage, which is partly mediated by its reactive nitrogen and oxygen species (RNOS) scavenger effect [1,32,33]. Among the flavonoids present in propolis extracts, pinocembrin has been linked to the inhibition of metalloproteinases (MMP) expression in cartilage, more precisely MMP-1, MMP-3, and MMP-13 [34]. MMPs are widely recognized for playing a key role in the breakdown of cartilage’s extracellular matrix throughout the progression of OA [2].

Portuguese propolis is far from being completely characterized but results to date indicate that this natural product exhibits some biological properties, namely antimicrobial, mainly antibacterial, but also antifungal activities [35,36,37,38,39]; antioxidant potential [35,40,41,42]; as well as genotoxic [40,41,43] and antitumor [38,44,45,46] activities, renewing the interest in researching national propolis. More recently, Portuguese propolis samples from Gerês revealed constancy in biological and chemical profiles over the years [35], which seems to be a distinctive and particular feature of this kind of propolis. Moreover, Peixoto et al. [36] reported that blends of five ethanol extracts of propolis from Gerês collected over five different years are equally or even more interesting than the individual extracts, and a step towards standardization since combining less and more active extracts resulted in comparable bioactivities. Similar results were obtained later by the same team while combining ethanol extracts of propolis collected from different apiaries and harvesting years [37], which further highlights the usefulness of propolis blends to increase the availability and to value this beehive resource. Overall, these studies support the great potential of propolis mixtures and increase the interest and the valorization of this natural product. At the same time, these data demonstrate the potential impact of Portuguese propolis on the development of innovative and bioactive propolis-based formulations. Thus, the main goal was to determine the effect of Portuguese propolis collected in Gerês as an anti-inflammatory agent in osteoarthritis. Antioxidant capacity was also determined since phenolics and flavonoid compounds of propolis contribute to the antioxidant activity and act as anti-inflammatory agents [47]. To the best of our knowledge, this is the first time that Portuguese propolis has been investigated for its capacity to reduce inflammation in vivo, whether as individual samples or mixtures. The in vitro anti-inflammatory potential was assessed through the inhibition of BSA denaturation assay, a methodology distinct from the one previously reported for other Portuguese propolis samples [41,48].

## 2. Materials and Methods

### 2.1. Propolis Samples

Propolis was collected in an apiary sited near the Cávado River, between Paradela and Sirvozelo, in Montalegre, Gerês, Portugal (41°45′41.62″ N; 7°58′03.34″ W). Raw samples were harvested over a 5-year period (2011–2015) and identified with the capital letter G (referring to its provenance: Gerês) followed by the last two digits of the harvesting year, namely G11, G12, G13, G14, and G15. Propolis samples were kept at 4 °C until use.

### 2.2. Preparation of Propolis Hydroalcoholic Extracts

Hydroalcoholic extraction of raw propolis was performed to achieve a final stock concentration of 80 mg/mL following the procedure described by Freitas et al. [35] and taking into consideration the yields previously obtained for the extraction of each sample with ethanol 70% (A.S. Freitas, personal communication). Briefly, 10 mL of ethanol (Analytical grade ACS, Scharlau, Barcelona, Spain) 70% (*v*/*v*) was added separately to 2.68 g of G11, 2.75 g of G12, 2.63 g of G13, 2.53 g of G14, and 2.67 g of G15, and each mixture was maintained under orbital agitation, in the dark, at 100 rpm for 24 h at 25 °C. Then, each propolis suspension was centrifuged for 5 min at 5000 rpm and 4 °C, and the resultant supernatant was collected and reserved. The pellets were extracted again under the same conditions with 10 mL of ethanol 70%. After the second centrifugation, the resultant supernatant was mixed with the previously reserved one, generating stock solutions of 80 mg/mL of each G.EE_70_. Additional hydroalcoholic extracts were prepared with a final concentration of 40 mg/mL by diluting G.EEs_70_ in water (1:1), generating the G.EEs_35_. To distinguish the ten G.EEs, the percentage of the solvent was also added to each code: G11.EE_70_ and G11.EE_35_; G12.EE_70_ and G12.EE_35_; G13.EE_70_ and G13.EE_35_; G14.EE_70_ and G14.EE_35_; G15.EE_70_ and G15.EE3_35_.

A similar methodology was followed to prepare a hydroalcoholic extract of the mixture (m) of all the Gerês propolis samples (mG). Briefly, approximately 6.70 g of G11, 6.88 g of G12, 6.57 g of G13, 6.57 g of G14, and 6.67 g of G15 were incubated together with 125 mL of ethanol 70% (*v*/*v*), and this mixture was stirred orbitally at 100 rpm for 24 h, in the dark. After centrifugation and re-incubation under the same conditions, the resultant supernatant was mixed with the stored one, and a stock solution of 80 mg/mL of mG.EE_70_ was obtained. An additional mixture was prepared by diluting mG.EE_70_ in water (1:1), generating the mG.EE_35_.

### 2.3. Analysis of Total Polyphenols and Flavonoids Content

Determination of total phenolic content (TPC) was performed according to the Folin-Ciocalteu colorimetric method [48,49]. A volume of 10 µL of propolis sample (10–200 g/mL) was mixed with 50 µL of the Folin-Ciocalteu reagent (1:10) (Sigma-Aldrich, Lisbon, Portugal) and 40 µL sodium carbonate (Na_2_CO_3_) (Acros Organics, Porto, Portugal) 7.5% (*w*/*v*). Each mixture was incubated for 1 h at room temperature and the absorbance was measured at 760 nm. A gallic acid (GA) (Sigma-Aldrich, Lisbon, Portugal) solution (1–20 μg/mL) was used as standard, and results were expressed in gallic acid equivalents per milligram of G.EE (μg GAE/mg extract). 

Total flavonoid content (TFC) was determined as described by Woisky & Salatino [50], with some modifications. Briefly, a volume of 50 µL of each G.EE (100–1400 µg/mL) was mixed with aluminum chloride (AlCl_3_) (Acros Organics, Porto, Portugal) 2% (*w*/*v*) ethanol solution. The mixture was incubated for 1 h, at room temperature, and the absorbance was measured at 420 nm. A quercetin ethanol solution (5–200 µg/ mL) was used as standard. Results were expressed in quercetin (Acros Organics, Porto, Portugal) equivalents (QE) per milligram of G.EE (µg QE/mg extract).

### 2.4. Evaluation of Antioxidant Potential of Propolis Extracts

#### 2.4.1. DPPH Radical Scavenging Activity

The propolis scavenging activity was determined using the DPPH (2,2-diphenyl-2-picry-lhydrazyl) colorimetric method [35,51]. Briefly, 100 µL of an ethanol solution of DPPH• (Sigma-Aldrich Portugal, Lisbon, Portugal) 0.004% (*w*/*v*) was added to 50 µL of the propolis sample, to yield final concentrations ranging from 1 to 50 µg/mL. Then, these mixtures were incubated at room temperature, in the dark, for 20 min. Control was prepared with DPPH• and ethanol. Absorbance was measured at 517 nm, using ethanol as a blank. The EC_50_ (µg/mL), corresponding to the concentration of an extract needed to scavenge 50% of the initial DPPH•, was calculated and expressed as the mean value of three independent experiments carried out in triplicate. GA was used as standard.

#### 2.4.2. Superoxide Anion Radical Scavenging Capacity 

The propolis capacity to scavenge the oxygen radical (O_2_^•−^) was evaluated using a non-enzymatic reaction [52,53]. Propolis extracts were diluted in phosphate buffer (KH2PO4; Fisher Scientific, Porto, Portugal) at 19 mM, pH 7.4, in order to obtain a concentration range between 75 and 300 µg/mL. A volume of 50 µL of this solution was mixed with 50 µL of NADH 166 µM, 150 µL of NBT 43 µM, and 50 µL of PMS 2.7 µM (VWR, Carnaxide, Portugal). This mixture was incubated at room temperature for 5 min and the absorbance was measured at 560 nm in a microplate reader (SpectraMax Plus, Wokingham, UK). Control was prepared substituting G.EEs with phosphate buffer. An additional control, also nominated blank solution, was carried out with PMS and NADH. Since greater O2^•−^ scavenging activity is shown by a decrease in absorbance of the reaction mixture, the scavenging activity was estimated as the percentage of reduction. The EC_50_ (µg/mL) was calculated and expressed as the mean value of three independent assays with triplicates each.

### 2.5. Evaluation of Propolis Anti-Inflammatory Potential

#### 2.5.1. In Vitro Anti-Inflammatory Assay: Inhibition of BSA Denaturation

The in vitro anti-inflammatory potential of propolis from Gerês was determined using the inhibition of heat-induced bovine serum albumin (BSA) denaturation technique [54,55], with some minor modifications. Briefly, the propolis samples, namely G.EEs and the mG.EE, were dissolved in phosphate buffer 0.066 M, pH 5.3, at 27 °C and mixed with equal amounts of BSA (Sigma-Aldrich Portugal, Lisbon, Portugal) 0.16% (*w*/*v*) in the same buffer. Then, the mixture was incubated at 70 °C for 35–40 min and the absorbance was measured at 594 nm. Negative and positive controls were made with phosphate buffer and acetylsalicylic acid (ASA) or aspirin, a known non-steroid anti-inflammatory drug (NSAID), respectively. The percentage of inhibition of precipitation (albumin denaturation) by each propolis sample was determined and expressed as the mean value of three independent assays with triplicates each.

#### 2.5.2. In Vivo Anti-Inflammatory Assay: Osteoarthritis Model

##### Animals and Ethical Issues

The in vivo anti-inflammatory assay was performed using adult male Wistar Han rats (*n* = 13, Charles Rivers, Barcelona, Spain), weighing between 350 and 450 g. Pairs of animals were housed together in a properly sanitized and well-ventilated room, under standard conditions: 12 h light-dark cycle (beginning at 8:00 a.m.), temperature ranging between 20–24 °C, and relative humidity of 55 ± 10%. The animals had access to a standard diet (food and water ad libitum), with their health status being checked weekly by the on-site veterinarian. Before the experiment, all animals were subjected to work conditions (handling and experimental room environment) to be adapted to the surroundings. The Institutional Ethical Commission of the ICVS gave its approval to all experimental methodologies, and they all complied with the European Community Council Directives 86/609/EEC and 2010/63/EU regarding the use of animals for scientific purposes. Precautions were taken during the trials to minimize animal suffering and the number of animals used.

##### Induction of Osteoarthritis

The arthritis model applied in this study was the Kaolin-Carrageenan-Induced Arthritis model (C/K) (Knee) [56,57]. Briefly, after being anesthetized, animals were intraarticularly injected in the synovial cap of the right knee joint with a mixture of 3% (*w*/*v*) kaolin and 3% (*w*/*v*) carrageenan (Sigma-Aldrich, St. Louis, MO, USA) dissolved in sterile saline solution (0.9% NaCl) (final volume of 0.1 mL). At this point, the OA was induced. After the injection, twenty flexion and extension movements were performed for about 2 min.

Animals were anesthetized prior to OA induction with an intraperitoneal injection of a mixture of ketamine (0.75 mg/kg Imalgene^®^, Merial, Lisbon, Portugal)—an N-methyl-D-aspartate receptor antagonist—and medetomidine (0.5 mg/kg Dorbene^®^, ESTEVE, Carnaxide, Portugal)—an α-2 adrenergic receptor agonist. After OA induction, the anesthesia was reversed using atipamezole (1 mL/kg Antisedan^®^, Pfizer, Seixal, Portugal)—an α-2 adrenergic receptor antagonist. Animals were monitored until they were fully recovered (feeding and grooming).

##### Propolis Administration

The mG.EE_35_ was administrated to rats (100 mg/kg) once a day by gavage [58,59,60], the method adopted to simulate clinical administration in humans. Briefly, this process involves inserting a gastric gavage needle with a ball tip (16-gauge, 100 mm long), coupled to a 10 mL syringe, into the mouth and esophagus of an immobilized animal that is kept in an upright position.

**Figure 1:**
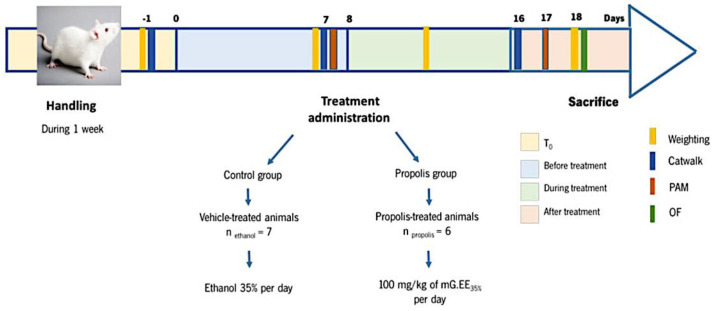
Schematic Representation of the Experimental Design. One week before OA induction with the Kaolin-Carrageenan-Induced Arthritis model, animals were handled daily to habituate to the experiment room conditions and the surroundings. Animals’ weight was assessed every Monday as an indirect indicator of health status. Propolis administration started one week after OA induction (T_0_) and was accomplished for one week (T_8_–T_18_). PAM was performed one week after OA induction (before treatment) and 10 days after propolis administration (after treatment). The catwalk was conducted one day before the OA induction (T_0_), one week after OA induction (before treatment), and, lastly, one week after propolis administration (after treatment). The Open Field (OF) test was performed at the end of the experiments before the animals were sacrificed.

Animals were divided into two different groups: a control group receiving the vehicle—1 mL of ethanol 35% (*v*/*v*) prepared by dilution of absolute ethanol (vehicle-treated animals; *n* = 7)) and the treatment group receiving propolis—1 mL of mG.EE_35_ (propolis-treated animals; *n* = 6). Figure 1 shows the experimental design and its scheduling. Animals’ weight was assessed every Monday. OA was induced in all animals as described on day 0. Daily administration of mG.EE35 by gavage was initiated on day 8 and the animals were sacrificed after 10 days of treatment (day 18). The pressure application measurement (PAM), the catwalk method, and the open field (OF) test were used to determine the effects of propolis on the behavior of the OA model.

##### Behavior Analysis of Propolis’ Effects on the OA Model

The pressure application measurement (PAM), the catwalk method, and the open field test were used to determine the effects of propolis on the behavior of the OA model.

Mechanical Hyperalgesia

The pressure application measurement (PAM) (Unit model 38500, Ugo Basile, Comerio, Italy) is a novel behavior technique that allows the direct measure of the hypersensitivity of joints [57,61]. A gradually increasing squeeze is applied across the knee joints of rats until the animal gave an indication of pain or discomfort by hind paw withdrawal or vocalization (behavioral response) [61,62]. 

After safely restraining the animal, the force transducer coupled to the recording base unit was positioned on one side of the knee joint, and the operator’s finger was positioned on the opposite side. The measuring range of this instrument varies between 0 and 1500 g. When a behavioral response was observed, the force transducer was immediately removed and the peak gram force (gF) applied was recorded as the limb withdrawal threshold (LWT) [62].

Gait Analysis—Catwalk Method

The analyses of changes in animals’ gait were assessed using the catwalk method [63,64,65,66], a technique where the animal is placed at the beginning of a walkway and must cross it to return to the home cage [64]. Briefly, the animals were individually placed in a black walkway measuring 100 × 7.6 cm, whose floor was lined with a 7.6 cm width white strip of paper (Roll Staples Auto 76 × 70 × 11, Braga, Portugal). Prior to the test, the animal’s fore and hind paws were painted with non-toxic red and blue dyes (GIOTTO Gouache, Scholar Giotto, France), respectively. After the test, the impairments in gait were analyzed by measuring: (i) stride length (SL); (ii) lateral distance between the two hind paws in successive steps (LD); (iii) box length (LP); (iv) box width (WP); (v) paw area (PA); (vi) distance between the first and the fifth hind toe (D1–5) and the (vii) distance between the second and the fourth hind toe (D 1–4) [63,66].

Anxiety-like Behavior—The Open Field Test

The open field (OF) test originally developed by Hall (1934) [67] to study emotionality in rodents allows the evaluation of several parameters, such as locomotor ability and anxiety-like behavior [57,67]. Each animal was placed in the testing arena -a grey square arena of 43.2 cm wide with the central area illuminated [58] and its behavior was video-recorded for 5 min [68], namely the distance traveled (in cm) and the time spent in the center zone. The number of feces left in the arena is another important and crucial parameter to assess anxiety-like behavior. The arena was cleaned with commercial ethanol 70% after each animal test.

##### Histological Analysis of the Knees

At the end of all experiments, a lethal dose of pentobarbitone (100 mg/kg, Eutasil^®^, CEVA, Algés, Portugal) was administered to the animals and perfused with 300 mL of a 4% (*m*/*v*) paraformaldehyde solution (PFA, Panreac, Barcelona, Spain) in 0.1 M PBS (phosphate buffered saline) (pH 7.4). The right knees were then excised and fixed in PFA 4% (*v*/*v*) for one week. Then, they were decalcified in a decalcifying solution (BiodecR, Bio-Optica, Milan Italy) for another week. The tissues were individually embedded in paraffin, and 4-µm serial sections were obtained at the medial levels in the sagittal plane. Lastly, the sections were stained with hematoxylin and eosin (HE) (Sigma-Aldrich, St. Louis, MO, USA) and examined under the microscope to check for any histopathologic abnormalities.

### 2.6. Statistical Analysis

Unless otherwise stated experiments were done at least three times, each with three replicates per treatment. All data are presented as means ± SD (standard deviation) or mean ± SEM (standard errors of the mean). The statistical analysis was performed using GraphPad Prism 7 software (GraphPad Software, Inc). Differences between results were evaluated using an ANOVA (one-way analyses of variance) followed by a t-test with Bonferroni correction for multiple comparisons. Only the analysis with a *p* < 0.05 were considered statistically significant (* 0.05 > *p* ≥ 0.01, ** 0.01 > *p* ≥ 0.001, *** *p* < 0.001).

## 3. Results

### 3.1. Total Polyphenol and Flavonoid Contents

**Figure 2:**
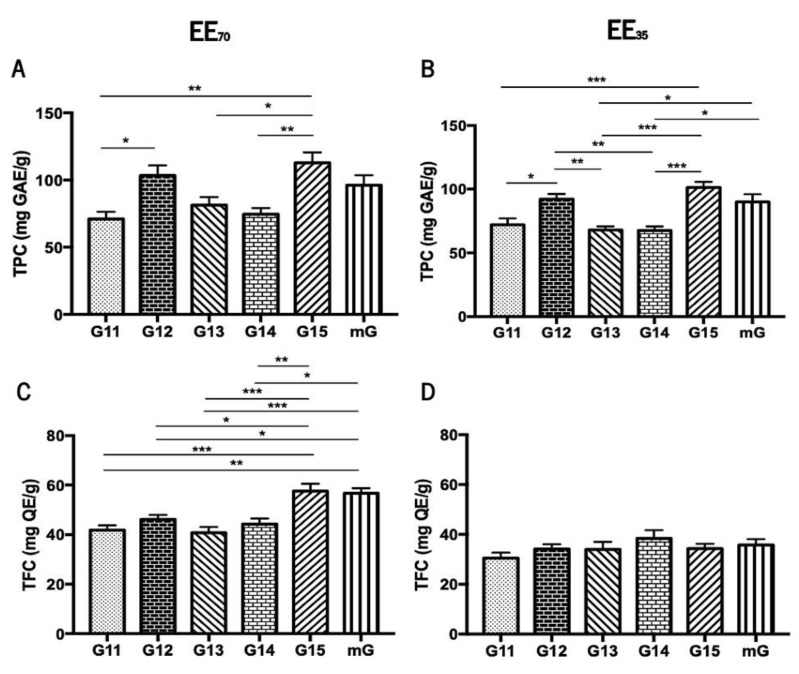
Total phenolic content (TPC) and total flavonoid content (TFC) of propolis from Gerês. TPC of G.EEs_70_ (**A**) and of G.EEs_35_ (**B**) measured in mg GAE/g; TFC of the G.EEs_70_ (**C**) and of G.EEs_35_ (**D**) measured in mg QE/g. Results are expressed as means ± SEM (* 0.05 > *p* ≥ 0.01, ** 0.01 > *p* ≥ 0.001, *** *p* < 0.001) (G: Gerês; mG: a mixture of all the propolis samples from Gerês harvested over 5 years; mg GAE/g: milligram gallic acid equivalents per gram of EE; mg QE/g: milligram quercetin equivalents per gram of EE).

The Folin-Ciocalteu colorimetric assay [48,49] and the Aluminium Chloride test [51] were used to evaluate the total phenolic content (TPC) and the total flavonoid content (TFC), respectively, of the five G.EEs, i.e., G11.EE, G12.EE, G13.EE, G14.EE, G15.EE, and of the two resultant mixtures mG.EEs, mG.EE_35_ and mG.EE_70_ (Figure 2). Overall, the TPC of propolis from Gerês ranged from 67.62 ± 7.68 to 112 ± 19.24 mg GAE/g whereas TFC varied between 30.49 ± 3.85 and 57.55 ± 6.69 mg GAE/g (Supplementary Table S1). Additionally, the TPC and TFC of the mG.EEs were higher than the average of the content values reported for all the individual samples of each EE group.

The total phenolic content demonstrated to be significantly different between samples harvested in different years (Figure 2A,B). More precisely, the post-hoc analysis for the group EE_70_ showed significant differences (ANOVA_1W_, F(5, 30) = 6.5, *p* < 0.001; Figure 2A) between (i) G11.EE_70_ and G12.EE_70_; (ii) G11.EE_70_ and G15.EE_70_; (iii) G13.EE_70_ and G15.EE_70_; and (iv) G14.EE_70_ and G15.EE_70_. Similarly, the same analysis for the G.EE_35_ group also demonstrated significant differences (ANOVA_1W_, F(5,30) = 10.9, *p* < 0.001; Figure 2B), namely (i) G11.EE_35_ and G12.EE_35_, (ii) G11.EE_35_ and G15.EE_35_; (iii) G12.EE_35_ and G13.EE_35_; (iv) G12.EE_35_ and G14.EE_35_; (v) G13.EE_35_ and G15.EE_35_; (vi) G13.EE_35_ and mG.EE_35_; (vii) G14.EE_35_ and G15.EE_35_; and (viii) G14.EE_35_ and mG.EE_35_. According to these analyses, we can establish a sequence in descending order of phenolic contents for each propolis sample: G15 ≥ G12 ≥ mG > G13 ≥ G14 ≥ G11 in the case of the EE_70_ group, and G15 ≥ G12 ≥ mG > G11 ≥ G13 = G14 for the EE_35_ group. In summary, the G15 has the highest concentration of polyphenols in both the EE_70_ and EE_35_ groups and the mixtures´ TPC values were either higher than those found for some individual extracts (G13, G14, and G15) or identical to the highest concentrations observed in the G15 and G12 extracts.

The total flavonoid content showed significant differences between the tested samples only in the EE_70_ group (Figure 2C,D) suggesting that the harvesting year affected the amount of flavonoids in this group. Post-hoc analyses of revealed significant differences (ANOVA_1W_, F(5,24) = 10.8, *p* < 0.001; Figure 2C) between (i) G11.EE70 and G15.EE70; (ii) G11.EE70 and mG.EE70; (iii) G12.EE70 and G15.EE70; (iv) G12.EE70 and mG.EE70; (v) G13.EE70 and G15.EE70; (vi) G13.EE70 and mG.EE70; (vii) G14.EE70 and G15.EE70; (viii) G14.EE70 and mG.EE70. A similar sequence of the propolis samples of the EE_70_ group in terms of TFC is the following: G15 = mG > G12 ≥ G14 ≥ G11 ≥ G13. Although no significant differences were observed, a sequence ordering of flavonoid content could be proposed for the EE_35_ too, namely G14 ≥ mG ≥ G15 = G12 = G13 ≥ G11. In short, G15/mG and G14 were the samples with the highest contents in flavonoids in EE_70_ and EE_35_ groups, respectively. TFC results reinforce the previous results regarding the TPC. 

### 3.2. Antioxidant Activity of Propolis 

**Figure 3:**
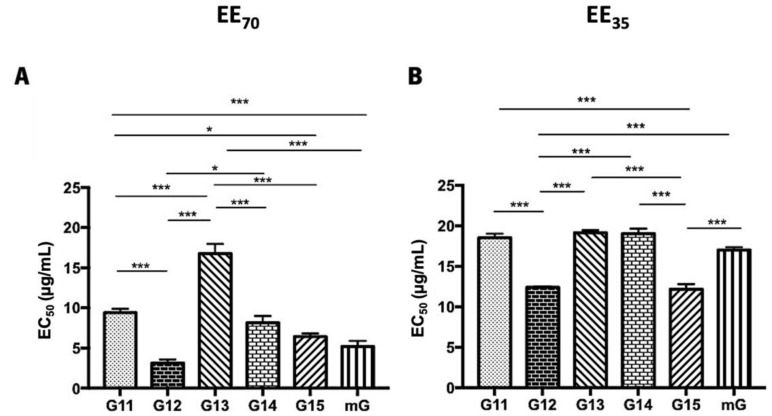
DPPH scavenging capacity of propolis hydroalcoholic extracts from Gerês. Propolis hydroalcoholic extracts have ethanol 70% (*v*/*v*) (EE_70_)—(**A**) or 35% (EE_35_)—(**B**). Results are expressed in EC_50_ (µg/mL) as mean ± SEM. (* 0.05 > *p* ≥ 0.01, *** *p* < 0.001) (DPPH: 2,2-diphenyl-2-picry-lhydrazyl; G: Gerês; mG: a mixture of propolis samples).

DPPH• radical scavenging and superoxide anion scavenging assays were used to evaluate the antioxidant capacity of propolis hydroalcoholic extracts from Gerês and the resultant mixtures (mG) (Figure 3 and Supplementary Table S2). Globally, the EE_70_ group presented lower EC_50_ values than the EE_35_ group. The lowest EC_50_ values were displayed by the G12, G15, and mG extracts, both in the EE_70_ and EE_35_ groups. However, a distinct sequence can be found in each group in terms of the analyzed scavenging activity.

The post-hoc analyses, in the G.EE_70_ group (Figure 3A), showed significant differences (ANOVA_1W_, F(6,13) = 57.6, *p* ≤ 0.001; Figure 3A) between the EC_50_ of (i) G11.EE_70_ and G12.EE_70_, (ii) G11.EE_70_ and G13.EE_70_, (iii) G11.EE_70_ and G15.EE_70_, (iv) G11.EE_70_ and mG.EE_70_, (v) G12.EE_70_ and G13.EE_70_, (vi) G12.EE_70_ and G14.EE_70_, (vii) G13.EE_70_ and G14.EE_70_, (viii) G13.EE_70_ and G15.EE_70_, and (ix) G13.EE_70_ and mG.EE_70_. Thus, the antioxidant sequence of G.EE_70_ is G12 = mG = G15 > G14 ≥ G11 ˃ G13. On the other hand, in the G.EE_35_ group (Figure 3B), the same analysis revealed significant differences (ANOVA_1W_, F(6,19) = 231, *p* ≤ 0.001; Figure 3B) between (i) G11.EE_35_ and G12.EE_35_, (ii) G11.EE_35_ and G15.EE_35_, (iii) G12.EE_35_ and G13.EE_35_, (iv) G12.EE_35_ and G14.EE_35_, (v) G12.EE_35_ and mG.EE_35_, (vi) G13.EE_35_ and G15.EE_35_, (vii) G14.EE_35_ and G15.EE_35_, (viii) G15.EE_35_ and mG.EE_35_. Thus, the following sequence can be proposed for the antioxidant capacity of G.EE_35_, namely G12 = G15 > mG ≥ G11 ≥ G13 = G14. This way, and in general, mixtures exhibited higher antioxidant potential than almost all the individual extracts, indicating that blending propolis with either a high or low antioxidant capacity may enhance this bioactivity.

**Figure 4:**
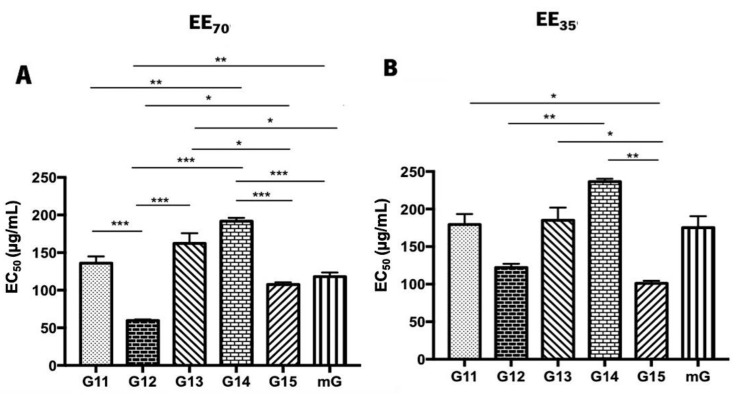
Superoxide anion scavenging activity of propolis hydroalcoholic extracts from Gerês. Propolis hydroalcoholic extracts have ethanol 70% (*v*/*v*) (EE_70_)—(**A**) or 35% (EE_35_)—(**B**). Results are expressed in EC_50_ (µg/mL) as mean ± SEM. (* 0.05 > *p* ≥ 0.01, ** 0.01 > *p* ≥ 0.001, *** *p* < 0.001) (G: Gerês; mG: a mixture of propolis samples).

The superoxide anion scavenging assay was also used to assess the antioxidant potential of propolis hydroalcoholic extracts from Gerês [69] (Figure 4 and Supplementary Table S3). Similar to the DPPH• assay, G.EEs_70_ exhibited lower EC_50_ values than the G.EEs_35_. In both groups, G12, G15, and mG show increased antioxidant activities, but, again, different patterns of antioxidant capacity can be established for each group considering the post-hoc analysis. For the G.EE_70_ group, EC_50_ values were significantly different (ANOVA_1W_, F(5,9) = 38.8, *p* ≤ 0.001; Figure 4A) between (i) G11.EE_70_ and G12.EE_70_, (ii) G11.EE_70_ and G14.EE_70_, (iii) G12.EE_70_ and G13.EE_70_, (iv) G12.EE_70_ and G14.EE_70_, (v) G12.EE_70_ and G15.EE_70_, (vi) G12.EE_70_ and mG.EE_70_, (vii) G13.EE_70_ and G15.EE_70_, (viii) G13.EE_70_ and mG.EE_70_, (ix) G14.EE_70_ and G15.EE_70_, (x) G14.EE_70_ and mG.EE_70_. The G.EE_35_ post-hoc analysis revealed significant differences (ANOVA_1W_, F(5,6) = 18.5, *p* = 0.001; Figure 4B) between (i) G11.EE_35_ and G15.EE_35_, (ii) G12.EE_35_ and G14.EE_35_, (iii) G13.EE_35_ and G15.EE_35_, and (iv) G14.EE_35_ and G15.EE_35_. In light of these results, we may establish a sequence of superoxide anion scavenging capacity, for both EE_70_ and EE_35_ groups: G12 > G15 = mG ≥ G11 ≥ G13 ≥ G14 and G15 ≥ G12 = mG = G11 ≥ G13 ≥ G14, respectively. Overall, these findings are consistent with the DPPH• results and strengthen the potential of the mixtures. 

### 3.3. Anti-Inflammatory Potential In Vitro of Propolis 

**Figure 5:**
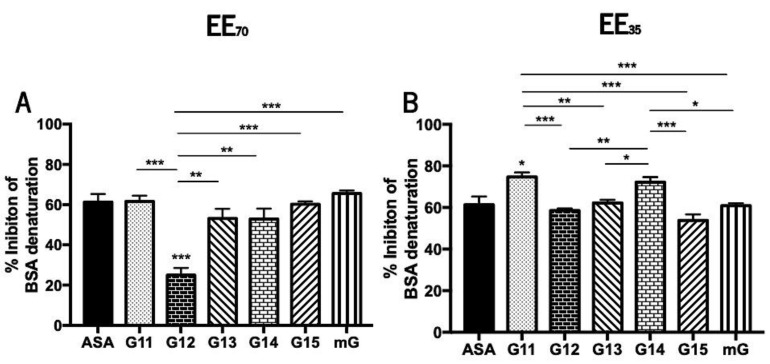
Capacity of G.EEs_70_ (**A**) and G.EEs_35_ (**B**) to inhibit heat-induced BSA denaturation. Data presented as mean ± SEM (* 0.05 > *p* ≥ 0.01, ** 0.01 > *p* ≥ 0.001, *** *p* < 0.001). (G: Gerês; mG: a mixture of propolis samples from Gerês; ASA: acetylsalicylic acid).

During denaturation, proteins lose their structure and, consequently, their biological functions [70]. This loss of function is well known during the inflammatory process [69,70,71] so the anti-inflammatory effect of natural compounds can be studied in vitro evaluating its capacity to reverse such biochemical process. Propolis’ anti-inflammatory potential was evaluated in vitro by measuring its capacity to inhibit the heat-induced denaturation of the BSA protein (Figure 5 and Supplementary Table S4). 

Curiously and contrary to the tendency observed for the antioxidant potential, G.EEs_35_ exhibited higher inhibition of BSA denaturation than the corresponding samples in the EE_70_ group. Nevertheless, G14 is the propolis sample with the highest anti-inflammatory capacity (Supplementary Table S4) regardless the extraction solvent. The mixtures outperformed almost every individual G.EE_35_, as well as G12 and G15 in the case of the EE_70_ group, in terms of anti-inflammatory efficacy (Supplementary Table S4).

Anti-inflammatory activity showed to be significantly different between samples harvested in different years (Figure 5). More specifically, the post-hoc analysis for the EE_70_ group revealed significant differences (ANOVA_1W_, F(6, 14) = 14.5, *p* < 0.001; Figure 5A) between G12.EE and all the remaining extracts. All G.EEs_70_ except G12.EE demonstrated similar behavior and, consequently, similar anti-inflammatory capability when compared to acetylsalicylic acid (ASA; EC_50_ = 61.25 ± 6.93 µg/mL), the commercial anti-inflammatory used as standard. Moreover, in this group, G12.EE presented the lowest anti-inflammatory capacity and the following hierarchy of anti-inflammatory capacity can be established: mG ≥ G11 = G15 ≥ G13 = G14 > G12, indicating advantage of employing a propolis mixture as anti-inflammatory instead of a single propolis sample. 

Regarding the G.EEs_35_ group, some significant differences were also observed (ANOVA_1W_, F(6, 32) = 13, *p* < 0.001; Figure 5B). All of the G.EEs_35_ had identical anti-inflammatory capacity to ASA, except the G11.EE and G14.EE, which appeared to have stronger anti-inflammatory potential, with the following sequence of % of inhibition of BSA denaturation: G11 ≥ G14 > G13 = mG ≥ G12 ≥ G15, once again showing that combining propolis samples with various anti-inflammatory potential can strengthen this effect.

### 3.4. Anti-Inflammatory Capacity In Vivo of Propolis 

Considering the previous results obtained regarding the antioxidant potential, the anti-inflammatory effects in vitro, and the availability of propolis samples, mG.EE_35_ was the sample chosen for the in vivo assays.

#### 3.4.1. Propolis from Gerês Improves the Mechanical Hyperalgesia

**Figure 6:**
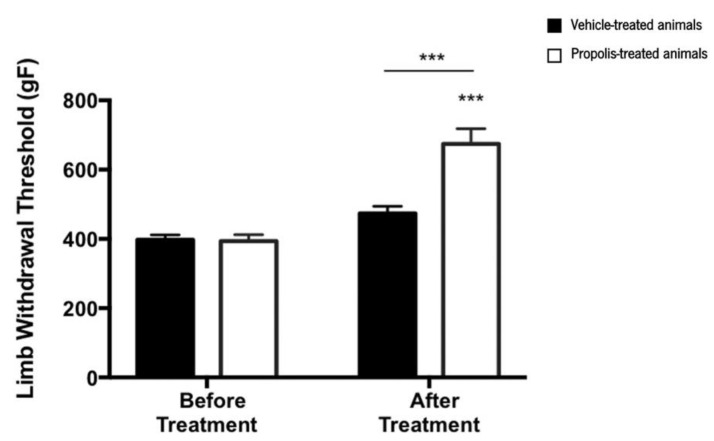
Evolution of the limb withdrawal threshold (LWT) in the right knee of ethanol (vehicle) and propolis-treated animals. The LWT was measured using the PAM test and the results were expressed as mean ± SEM (*** *p* < 0.001; *** above the bar is related to before the treatment of the same tested group) (■—vehicle-treated animals; □—propolis-treated animals).

Results from the pressure application measurement (PAM) test indicated that the limb withdrawal threshold (LWT) was significantly changed over the course of the trial (ANOVA_2W_, interaction between group and time, F(1,15) = 17.1, *p* < 0.001; Figure 6). Indeed, LWT significantly increased after treatment compared to the time point before the treatment (ANOVA_2W_, main effect of time F(1,15) = 51.9, *p* < 0.001; Figure 6), an effect that varied with the experimental group (ANOVA_2W_, main effect of group F(1,15) = 15.8, *p* = 0.001; Figure 6). This significant increase in the LWT was only verified in propolis-treated animals (not in the vehicle-treated ones), suggesting that propolis from Gerês influences the mechanical hyperalgesia of the OA-induced model.

#### 3.4.2. Propolis from Gerês Enhances Animal’s Gait

**Figure 7:**
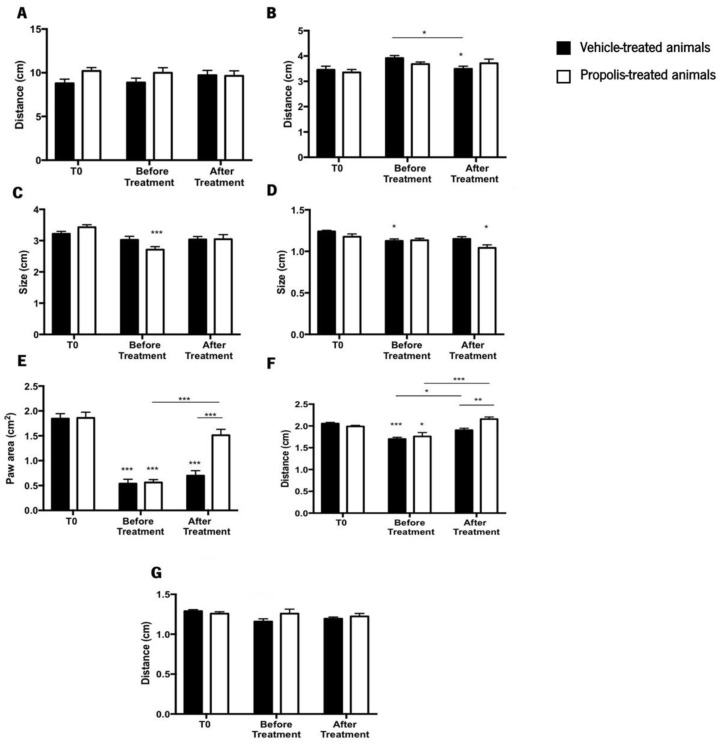
Evaluation of the animal’s gait by the catwalk method. The parameters evaluated in this study were (**A**) stride length (SL); (**B**) lateral distance between two hind paws in successive steps (LD); (**C**) box length (LP); (**D**) box width (WP); (**E**) paw area (PA); (**F**) distance between the first and the fifth hind toe (D1–5); and (**G**) distance between the second and the fourth hind toe (D 1–4). Results were expressed as mean ± SEM (* 0.05 > *p* ≥ 0.01, ** 0.01 > *p* ≥ 0.001, *** *p* < 0.001; *** above the bar is related to the T_0_ of the same tested group) (■—vehicle-treated animals; □—propolis-treated animals).

The catwalk approach was used to investigate how propolis treatment affected the gait pattern of an OA-induced rodent model, along with histological analysis. This study is crucial and essential to understand how osteoarthritis is progressing [72,73]. The parameters evaluated were the stride length (SL), the lateral distance between two hind paws in successive steps (LD), the box length (LP), the box width (WP), the paw area (PA), the distance between the first and the fifth hind toe (D 1–5), and the distance between the second and the fourth hind toe (D 1–4) (Figure 7).

Regarding the SL, no discernible differences were observed between the treatment groups throughout the experiment (ANOVA_2W_, main effect of the interaction between the time and the group: F(2,84) = 1.08, *p* = 0.34; Figure 7A). The same pattern was noticed in the LD (ANOVA_2W_, main effect of the interaction between the time and the group: F(2,84) = 1.76, *p* = 0.18; Figure 7B), although this parameter for the vehicle-treated animals after treatment was significantly lower than at T_0_ and before treatment (ANOVA2W, main effect of time F(2,84) = 5.11, *p* = 0.008; Figure 7B). Considering the LP results, no significant differences were detected during the course of the study (ANOVA_2W_, main effect of the interaction between the time and the group: F(2,87) = 3.31, *p* = 0.05; Figure 7C). Nevertheless, the LP of propolis-treated animals decreased significantly before treatment when compared to the T_0_ (ANOVA_2W_, main effect of time F(2,87) = 9.23, *p* < 0.001; Figure 7C) but recovered after treatment.

The WP parameter’s statistical results revealed that no statistically significant differences existed during the experimental period (ANOVA_2W_*,* main effect of the interaction between the time and the group: F(2,87) = 2.36, *p* = 0.1; Figure 7D). However, compared to the T_0_, the WP of vehicle-treated animals before treatment significantly decreased. Additionally, propolis-treated animals showed a significant decline in the WP parameter after treatment, in comparison to the time point T_0_ (ANOVA_2W_, main effect of time F(2,87) = 8.7, *p* < 0.001; Figure 7D). Contrary to the tendency observed in the previously evaluated parameters, significant differences were obtained in the PA during the trial (ANOVA_2W_, main effect of the interaction between the time and the group: F(2,21) = 10.3, *p* < 0.001; Figure 7E). Compared to T_0_, the PA decreased significantly before treatment, a tendency that was reversed after propolis treatment (ANOVA_2W_, main effect of time: F(2,21) = 83.1, *p* < 0.001; Figure 7E). This effect changed during the course of the experiment (ANOVA_2W_, main effect of group: F(2,21) = 11.7, *p* = 0.003; Figure 7E). Furthermore, the post-hoc analysis revealed a decrease in the PA after OA induction, a condition that was reversed by propolis treatment.

D1–5 parameter exhibited the same pattern of the PA parameter, being significantly influenced by trial time (ANOVA_2W_, main effect of the interaction between time and group F(2,54) = 6.24, *p* = 0.004; Figure 7F). D1–5 decreased in a significant way before treatment compared to T_0_, however, it recovered after treatment. This effect changed throughout the experiment (ANOVA_2W_, main effect of group F(1,54) = 4.87, *p* = 0.03; Figure 7F). Post-hoc analysis demonstrated that the induction of OA lead to a decrease in D1–5 (before treatment time point). After treatment, this distance increased for propolis-treated animals, but not in the vehicle-treated animals (Figure 7F). Regarding D 2–4, no significant differences were observed during the trial course (ANOVA_2W_, main effect of the interaction between the time and the group: F(2,85) = 2.04, *p* = 0.14; Figure 7G).

Considering all the previous results, we may conclude that propolis influences the gait parameters of OA-induced animals by expanding the paw area and improving the distance between the first and fifth toe.

#### 3.4.3. Propolis from Gerês Decreased Histological Severity of OA-Induced Animals

**Figure 8:**
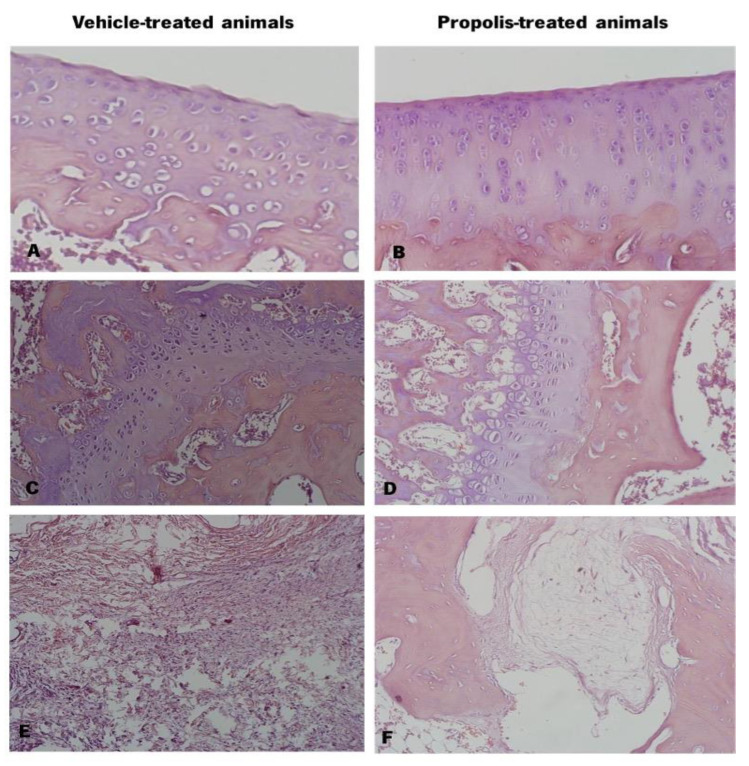
Histology of the knee joint of vehicle and propolis-treated animals stained with hematoxylin-eosin. (**A**) Knee joint cartilage with surface erosion and altered chondrocytes arrangement; (**B**) Knee joint cartilage with organized chondrocytes columns; (**C**) Alveolar bone with thickening of the epiphyseal line and trabeculae; (**D**) Alveolar bone; (**E**) Periarticular tissues with oedema and high level of inflammatory response; (**F**) Periarticular tissue with oedema and less pronounced inflammatory response.

The excised knee joints of vehicle and propolis-treated animals were stained with hematoxylin-eosin (Figure 8). Observing the cartilage, the vehicle-treated animals exhibited a deformed and altered chondrocyte arrangement and some cartilage surface erosion (Figure 8A). Contrarily, the chondrocytes of propolis-treated animals were displayed in very well-organized columns (Figure 8B). At the bone level, vehicle-treated animals (Figure 8C) showed a thickening of trabeculae in the alveolar bone and of the epiphyseal line, compared to propolis-treated animals (Figure 8D). Regarding the joint tissues, both experimental groups revealed oedema in periarticular tissues (Figure 8E,F). However, the corresponding anti-inflammatory response was substantially less pronounced in the propolis-treated group (Figure 8F).

#### 3.4.4. Propolis from Gerês Did Not Reverse the Anxiety-Like Behavior of OA-Induced Animals

Anxiety is a psychological disorder that can be detected when animals are confronted with new challenges/problems [74]. Several methods can be used to measure anxiety-like behavior but the OF test was the one selected because it is a quick, easy assay, and it is the only standardized test for evaluating locomotor ability [74,75]. Thus, to assess the effectiveness of propolis administration in reversing the OA-induced anxiety-like behavior in animals, data on the time spent in the periphery versus in the center of the arena were recorded in the OF test, as well as the number of fecal boils left in the arena at the end of the trial. Generally, the anxious-like animals tend to spend more time in the periphery of the arena and leave more fecal boils. 

**Figure 9:**
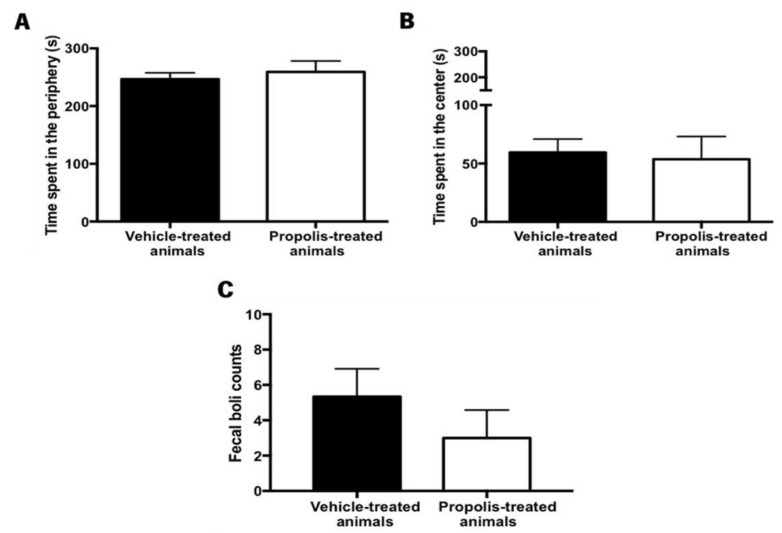
Evaluation of the anxiety-like behavior after propolis administration in the vehicle and propolis-treated animals. This parameter was evaluated using the OF test. Time spent (**A**) in the periphery and (**B**) in the center of the OF arena by the vehicle and the propolis-treated animals; (**C**) Fecal boils left in the OF arena at the end of the experimental session by the same tested group of animals. Results were expressed as mean ± SEM. (■—vehicle-treated animals; □—propolis-treated animals).

No significant differences were detected between the experimental groups in what concerns the time spent in the periphery (t_5.08_ = 0.58, *p* = 0.59; Figure 9A) and the center of the arena (t_0.258_ = 3.41, *p* = 0.81; Figure 9B). Accordingly, the total number of fecal boils left in the arena was not significantly different between the experimental tested groups (t_0.996_ = 8, *p* = 0.3483; Figure 9C). However, in this case, a decreased tendency could be observed.

The impact of propolis on animal’s locomotor ability was also assessed, by calculating the total distance traveled in the open field test (OF). However, no significant differences were observed between the vehicle and the propolis-treated animals (t_1.72_ = 6.95, *p* = 0.13), revealing that propolis did not improve the locomotor ability of OA-induced animals.

## 4. Discussion

Osteoarthritis, a chronic and degenerative joint disease, is the second leading cause of disability in the world [1,2,3,4]. Despite all the advances and research over the last years, none of the proposed strategies has been effective in generating functional and long-lasting tissue [1,14]. Due to the high prevalence of OA and the urgent need for an effective treatment, natural products, such as propolis compounds, have the potential to be used as an anti-inflammatory treatment [16,17]. Anti-inflammatory bioactivity was already described for two Portuguese propolis samples in in vitro experiments [76,77]. Still, studies with propolis from Gerês, the sample that seems rather unique due to the constancy of their chemical and biological profiles [22], are lacking as well as in vivo evidence of the anti-inflammatory potential of any Portuguese propolis. Therefore, in this study, we evaluated for the first time the anti-inflammatory potential of propolis samples from Gerês harvested over five years (G11-G15), either individually or combined in a blend, and both in vitro and in vivo. By mixing propolis from different harvesting years we expect to contribute to overcome the limitations of its low yield increasing the available propolis for the market. Additionally, mixtures may be a sustainable strategy and a partial solution for the widely reported standardization challenge of propolis [78].

Propolis’ chemical composition is extremely complex and highly variable [18,20,21] and is characterized by a high concentration of phenolic compounds, with flavonoids and aromatic acids being the two major classes identified [20,78]. In this study, the total phenolic and flavonoid contents obtained for hydroalcoholic extracts of individual and of blends of propolis samples from Gerês (G11_70/35_ -G15_70/35_) ranged from 67.62 ± 7.68 to 112 ± 19.24 mg GAE/g and 30.49 ± 3.85 and 57.55 ± 6.69 mg QE/g, respectively (Figure 2; Supplementary Table S1). These results seem to corroborate prior research that characterized propolis chemical composition as a year-dependent parameter [79,80], but in the case of propolis from Gerês such seasonal chemical variations are mainly quantitative and did not alter the spectrum of the identified compounds [35]. Peixoto et al. [36] reported TPC and TFC values ranging from 143.0 to 212.2 mg GAE/g and 31.0 and 51.7 mg QE/g, respectively, for the ethanol extracts of G11 to G15 samples. Comparing to the TPC and TFC values herein obtained for the hydroalcoholic extracts of the same propolis samples, it is clear the influence of the extraction solvent: ethanol 70% and 35% in the present work and absolute ethanol in the work of Peixoto and co-workers [36]. A significant impact of ethanol concentration in the TFC and TPC values was also noticed by Oroian et al. [81], who correlated an increase in ethanol concentration from 40% to 80% with a 42.87% and 175% increase in the polyphenol and flavonoid contents, respectively. Other studies show the importance of the solvent choice: propolis aqueous extracts have even significantly lower contents of bioactive compounds than ethanol extracts [82,83]. Ethanol has been widely considered the best solvent for propolis extraction and 70% was proposed as the ideal concentration [81]. TPC and TFC of the hydroalcoholic extracts of propolis from Gerês, either of the individual extracts or its blends, fall within the range specified for both national and European samples [28,43,78] but are higher than the ones of Brazilian propolis samples [50], highlighting the influence of geographical origin, climate conditions, and plant sources.

The mixtures of Gerês propolis samples (mG.EEs) showed TPC and TFC higher than the average of the content values found in the hydroalcoholic extracts prepared from a single propolis sample, indicating that sample blending does not reduce the concentration of these chemical components (Figure 2; Supplementary Table S1). The nature of the methodologies for TFC and TPC estimation might explain these results because the occurring reactions can be affected by synergisms of the phenolic substances that are mixed. It is a well-known fact that the Folin-Ciocalteu colorimetric assay generates higher polyphenol values in mixtures than in individual extracts [84]. Propolis blends can potentially minimize the variation observed within samples harvested in distinct years by attenuating the differences between individual samples and minimizing the heterogeneity, contributing to propolis normalization [37]. This evidenced potential of blends can be very useful for propolis applications in several areas, including the food sector and medicine. 

According to Sheng et al. [85], a natural substance can be identified as a potential natural antioxidant if it can react with DPPH•, a stable nitrogen-centered free radical [52,86], causing a change in coloration from purple to yellow [85,86]. In the case of the superoxide radical scavenging assay, the natural compounds display antioxidant capacity if reduce nitro blue tetrazolium, revealing a purple formazan [87]. Previous studies classified Portuguese propolis from Gerês as a natural compound with antioxidant potential [35,40,41] based on DPPH assays. Propolis samples from Algarve presented EC_50_ values ranging between 27 to 29 µg/mL, depending on the season of sample collection [41]. Falcão et al. [78] reported that propolis samples from the north of Portugal presented the best EC_50_ values, ranging from 10–30 µg/mL. Additionally, propolis from Gerês (Supplementary Table S2; Figure 3) seems to be promising when compared to other worldwide samples, such as Chinese propolis (EC_50_ values of 31.83–32.35 µg/mL). So, based on this antioxidant assay, Gerês propolis is an attractive type for antioxidant applications. However, contrary to observed in the DPPH results, the evaluated G.EEs displayed weaker superoxide radical scavenging capacity (Supplementary Table S3; Figure 4) than propolis from Algarve (EC_50_ values of 35–36 µg/mL) and South Brazil (EC_50_ values ranging between 0.20 to 2.91 µg/mL) [41,49]. Differences between the EC_50_ values obtained in each method can be explained by the process of free radicals generation: the DPPH itself is the stable free radical, and well-known in the propolis investigation groups; while in the superoxide anion scavenging assay the free radical is generated by the reaction of phenazine methosulfate (PMS) and nicotinamide adenine dinucleotide (NADH) [88].

Propolis mixtures (mG.EEs) display DPPH• (Figure 3; Supplementary Table S2) and superoxide scavenging (Figure 4; Supplementary Table S3) activities similar to the individual samples with the highest antioxidant capacity. This result revealed that mixing propolis retains the best antioxidant effect, which in turn might be related to the higher flavonoid content [28]. In comparison with the results previously reported for Gerês propolis mixtures extracted with absolute ethanol (mG.EE_100_) (EC_50_ values ranging from 11.8 to 13.7 µg/mL), mG.EE_70_ presented a higher antioxidant capacity (EC_50_ = 4.73 µg/mL), which can be related to the ethanol concentration used during the extraction process [36,37]. Not only the extraction solvent itself, but also its concentration may interfere with the chemical composition and, consequently, with the biological properties, e.g., the antioxidant potential, as has been reported [21,28,87,89]. Thus, these results corroborate the previous statements regarding TPC/TFC contents and mixtures, reinforcing that mixtures are interesting for antioxidant applications in areas like the food industry as an antioxidant agent in meat/fish products (food preservation) and in fruit texture conservation for instances [90]. Raw propolis is considered a food supplement in almost of the countries worldwide, more available commercially in the form of capsules [91] but it can also be found in the formulation of several supplements, mainly in the form of hydroalcoholic extracts [92].

The anti-inflammatory potential of Portuguese propolis samples from Gerês was evaluated both in vitro and in vivo. Anti-inflammatory agents must be able to prevent protein denaturation, which is a hallmark of the inflammatory process [70]. G.EEs_35_ had a similar or even higher capacity to inhibit BSA denaturation than G.EEs_70_ (Figure 5; Supplementary Table S4), suggesting that dilution did not interfere with in vitro anti-inflammatory capacity, and the same inhibition of protein denaturation can be achieved with less amount of propolis. This conclusion is particularly important given the low productivity and scarcity of propolis. Using the hyaluronidase assay, Silva et al. [48] and Miguel et al. [41] showed other Portuguese propolis samples exhibiting anti-inflammatory capacity (% inhibition ranging between 53.76 and 75.79%), being the only two studies that reported an anti-inflammatory potential for Portuguese propolis. However, several plant extracts, namely from *Oxalis corniculata Linn*, *Wedelia trilobata* (L.) *Hitchc*, and *Enicostema axillare* displayed a capacity to inhibit BSA denaturation ranging from 48 to 86%, which is comparable to the mG.EEs and G.EEs results (Figure 5; Supplementary Table S4) [70,72,93]. These plant extracts are well-known anti-inflammatory products, and propolis from Gerês seems to be a potent anti-inflammatory agent as well, with an effect that is similar to aspirin (inhibition of 61.25%). These outcomes are valid for the mixtures too, supporting the product and the blend’s prospective usage.

As the hydroalcoholic extracts of propolis mixtures (mG.EEs), either with 35% or 70% ethanol, exhibited strong antioxidant and anti-inflammatory effects in vitro, and considering both the scarcity of propolis and the restrictions to ethanol content by the food industry (less than 70% of ethanol) [39], we decided to pursue the in vivo experiments with the diluted combination, specifically the mG.EE_35_. In order to confirm the in vitro anti-inflammatory activity, in vivo assays using the OA-induced model were carried out (see Section 3.4). Mechanical hyperalgesia was the first evaluated behavior parameter (Figure 6). Untreated animals exhibited mechanical hyperalgesia immediately after the OA induction, an effect reversed by propolis administration, as evidenced by the observed rise in the LWT. This increase can be associated with an attenuation of pain that may be related to a reduction of the inflammation response due to Gerês propolis, which previously revealed an anti-inflammatory effect in vitro. Rayiti et al. [94] reported that chrysin, a propolis compound, can attenuate neuropathic pain by ameliorating mechanical hyperalgesia (doses varying between 100 and 200 mg/Kg). Moreover, Park and Kahng [59] demonstrated that the administration of a Korean propolis extract at a dose of 100 mg/Kg per day led to a decrease in the pain sensation of the OA-induced model identical to aspirin. Our findings are in accordance with these previously described outcomes, renewing the statement that Gerês propolis decreases pain sensation. 

The animal gait was also evaluated (Figure 7). A decrease in the PA after OA induction was observed, being completely reversed upon propolis administration (Figure 7E). Additionally, D1–5 was improved after the delivery of mG.EE_35_ (Figure 7F). This improvement in the animal’s gait can be related to an enhancement in the knee extension, which can be explained by the propolis capacity to reduce the pain sensation, acting as an anti-inflammatory compound. Rao et al. [95] demonstrated that plant extracts from *Rauvolfia tetraphylla* L. have the capacity to reduce the edema in the knee joint by reducing the inflammatory response. Our results corroborate this idea as propolis administration showed an anti-inflammatory effect by improving gait parameters. The histopathological analysis of the knee joint (Figure 8) revealed a lower level of joint degradation (Figure 8A,B) and decreased inflammatory response in the joint tissues of mG.EE_35_-treated animals (Figure 8E,F). Knowing that anti-inflammatory potential can be associated with polyphenols, the capacity of Gerês propolis extract to partially prevent join degradation can be related to the high phenolic content previously observed (Figure 2B,D). mG.EE_35_-treated animals. Previous studies reported that an Italian propolis extract has the capacity to prevent cartilage degradation and this effect was associated with the phenolic content that conferred to propolis the anti-inflammatory activity [32]. Hu et al. [96] reported that ethanol extracts of propolis interact with the production of cytokines, preventing events that culminate with cartilage degradation, as observed herein.

Although administration of propolis improved the mechanical hyperalgesia and the gait parameters in general, for the locomotor ability no significant differences were reported between the two experimental groups. These results can be explained by the fact that rats are quadruped animals, and this condition allows animals to easily compensate the joint impairments [97].

Anxiety behavior is one of the mood comorbidities associated with osteoarthritis [62] and was evaluated through the open field test (Figure 9). Conflicting information has been published over the years. In fact, Ji et al. [98] described that anxiety-like behavior occurred after 24 h after induction of OA with K/C, while in a similar study, Amorim et al. [58] reported that animals exhibited an anxiety-like behavior four weeks post-induction. In our research, the anxious-like behavior could not be reversed by propolis treatment (Figure 9). For both experimental groups, vehicle and propolis-treated animals, the time spent in the center (Figure 9B) was lower than the time spent in the periphery (Figure 9A), and the fecal boil left in the arena were similar for both groups (Figure 9C). Thus, our work does not demonstrate the capacity of propolis to reverse the anxiety-like phenotype. However, this lack of propolis effect could be related to the time frame of the experimental design and the short time of administration (one week) that might have been insufficient to reverse this phenotype [58,98]. Considering these outcomes, the administration of propolis should be extended in future experiments. Overall, and looking at all in vivo results, it is possible to conclude that propolis is a potent anti-inflammatory agent. 

## 5. Conclusions

Portuguese propolis, namely the Gerês propolis type, displays a wide spectrum of bioactivities, making this natural product appealing for several applications, including the food industry, specifically as a food supplement. The G11–G15 propolis extracts and the resultant blends demonstrated a strong antioxidant effect, as well as an in vitro anti-inflammatory effect similar to aspirin. Regarding the in vivo anti-inflammatory experiments, mechanical hyperalgesia as well as the gait pattern parameters (namely paw area and the D1–5) were improved in propolis-treated animals. Furthermore, the administration of mG.EE_35_, one of the best hydroalcoholic extracts with concern to all the assessed properties and respective parameters, partially prevented the degradation of joint tissues. 

To summarize, we provided the first evidence for an in vivo anti-inflammatory activity of Portuguese propolis. Moreover, we suggest that the use of propolis blends rather than individual extracts can be a better and more efficient approach to take full advantage of the bioactive potential of propolis since mixtures contribute to propolis normalization and provide a more sustainable approach to fully use this scarce natural resource. Overall, we believe that this natural product should continue to be explored as a potential source of compounds with antioxidant and anti-inflammatory potential. 

## Data Availability

Data is contained within the article.

## Supplementary Materials

The following supporting information can be downloaded at: https://www.mdpi.com/article/10.3390/foods11213431/s1. Table S1: Total phenolic content (TPC) and total flavonoid content (TFC) of the hydroalcoholic extracts of propolis from Gerês (G.EEs)and the resultant mixtures (mG); Table S2: DPPH• scavenging activity of hydroalcoholic extracts of propolis from Gerês; Table S3: Superoxide anion scavenging activity of hydroalcoholic extracts propolis from Gerês; Table S4: Inhibition of heat-induced BSA denaturation by hydroalcoholic extracts of Gerês propolis.

## References

[1] Arias C., Saavedra N., Saavedra K., Alvear M., Cuevas A., Maria-Engler S.S., Abdalla D.S.P., Salazar L.A. (2019). Propolis reduces the expression of autophagy-related proteins in chondrocytes under interleukin-1β stimulus. Int. J. Mol. Sci..

[2] Zheng W., Tao Z., Cai L., Chen C., Zhang C., Wang Q., Ying X., Hu W., Chen H. (2017). Chrysin Attenuates IL-1β-Induced Expression of Inflammatory Mediators by Suppressing NF-κB in Human Osteoarthritis Chondrocytes. Inflammation.

[3] Malemud C.J., Islam N., Haqqi T.M. (2003). Pathophysiological mechanisms in osteoarthritis lead to novel therapeutic strategies. Cells Tissues Organs.

[4] Soares A.K.C., de Sousa Júnior A.D., Lorençoni M.F., de Castro J.A., de Araujo Porto F.V., Pessoa I.S., Silva M.V.T.e., Pereira A.C.H., de Souza Andrade Moraes F., de Andrade T.U. (2021). In vitro and in vivo anti-inflammatory activity and chemical composition of Renealmia petasites Gagnep. Inflammopharmacology.

[5] Robinson W.H., Lepus C.M., Wang Q., Raghu H., Mao R., Lindstrom T.M., Sokolove J. (2016). Low-grade inflammation as a key mediator of the pathogenesis of osteoarthritis. Nat. Rev. Rheumatol..

[6] Zhang H., Cai D., Bai X. (2020). Macrophages regulate the progression of osteoarthritis. Osteoarthr. Cartil..

[7] Sun X., Zhen X., Hu X., Li Y., Gu S., Gu Y., Dong H. (2019). Osteoarthritis in the middle-aged and elderly in china: Prevalence and influencing factors. Int. J. Environ. Res. Public Health.

[8] Jones G., Ding C., Scott F., Glisson M., Cicuttini F. (2004). Early radiographic osteoarthritis is associated with substantial changes in cartilage volume and tibial bone surface area in both males and females. Osteoarthr. Cartil..

[9] Sun K., Luo J., Jing X., Xiang W., Guo J., Yao X., Liang S., Guo F., Xu T. (2021). Hyperoside ameliorates the progression of osteoarthritis: An in vitro and in vivo study. Phytomedicine.

[10] Zhang Z., Huang C., Jiang Q., Zheng Y., Liu Y., Liu S., Chen Y., Mei Y., Ding C., Chen M. (2020). Guidelines for the diagnosis and treatment of osteoarthritis in China (2019 edition). Ann. Transl. Med..

[11] Mobasheri A., Kalamegam G., Musumeci G., Batt M.E. (2014). Chondrocyte and mesenchymal stem cell-based therapies for cartilage repair in osteoarthritis and related orthopaedic conditions. Maturitas.

[12] Bonnet C.S., Walsh D.A. (2005). Osteoarthritis, angiogenesis and inflammation. Rheumatology.

[13] Santangelo K.S., Nuovo G.J., Bertone A.L. (2012). In vivo reduction or blockade of interleukin-1β in primary osteoarthritis influences expression of mediators implicated in pathogenesis. Osteoarthr. Cartil..

[14] Lotz M.K., Caramés B. (2011). Autophagy and cartilage homeostasis mechanisms in joint health, aging and OA. Nat. Rev. Rheumatol..

[15] Wieland H.A., Michaelis M., Kirschbaum B.J., Rudolphi K.A. (2005). Osteoarthritis—An untreatable disease?. Nat. Rev. Drug Discov..

[16] Liberal J., Ferreira I.V., Cardoso E.O., Silva A., Bartolomeu A.R., Martins J., Santiago K.B., Conti B.J., Neves B.M., Batista M.T. (2016). Anti-Inflammatory Activity of the Honeybee Plant- Derived Products Honey, Pollen and Propolis. Chemistry, Biology and Potential Applications of Honeybee Plant-Derived Products.

[17] Azab A., Nassar A., Azab A.N. (2016). Anti-inflammatory activity of natural products. Molecules.

[18] Marcucci M.C. (1995). Propolis: Chemical composition, biological properties and therapeutic activity. Apidologie.

[19] Umthong S., Phuwapraisirisan P., Puthong S., Chanchao C. (2011). In vitro antiproliferative activity of partially purified Trigona laeviceps propolis from Thailand on human cancer cell lines. BMC Complement. Altern. Med..

[20] Silva-Carvalho R., Baltazar F., Almeida-Aguiar C. (2015). Propolis: A Complex Natural Product with a Plethora of Biological Activities That Can Be Explored for Drug Development. Evid.-Based Complement. Altern. Med..

[21] Sforcin J.M., Bankova V. (2011). Propolis: Is there a potential for the development of new drugs?. J. Ethnopharmacol..

[22] Sforcin J.M. (2016). Biological Properties and Therapeutic Applications of Propolis. Phyther. Res..

[23] Kasote D., Bankova V., Viljoen A.M. (2022). Propolis: Chemical diversity and challenges in quality control. Phytochem. Rev..

[24] Kujumgiev A., Tsvetkova I., Serkedjieva Y., Bankova V., Christov R., Popov S. (1999). Antibacterial, antifungal and antiviral activity of propolis of different geographic origin. J. Ethnopharmacol..

[25] Šturm L., Ulrih N.P. (2020). Advances in the Propolis Chemical Composition between 2013 and 2018: A Review. eFood.

[26] Huang S., Zhang C.P., Wang K., Li G.Q., Hu F.L. (2014). Recent advances in the chemical composition of propolis. Molecules.

[27] Rufatto L.C., dos Santos D.A., Marinho F., Henriques J.A.P., Roesch Ely M., Moura S. (2017). Red propolis: Chemical composition and pharmacological activity. Asian Pac. J. Trop. Biomed..

[28] Kumazawa S., Hamasaka T., Nakayama T. (2004). Antioxidant activity of propolis of various geographic origins. Food Chem..

[29] Banskota A.H., Tezuka Y., Kadota S. (2001). Recent progress in pharmacological research of propolis. Phyther. Res..

[30] de Almeida E.C., Menezes H. (2002). Anti-inflammatory activity of propolis extracts: A review. J. Venom. Anim. Toxins.

[31] Sforcin J.M. (2007). Propolis and the immune system: A review. J. Ethnopharmacol..

[32] Cardile V., Panico A., Gentile B., Borrelli F., Russo A. (2003). Effect of propolis on human cartilage and chondrocytes. Life Sci..

[33] El-Ghazaly M.A., Abd El-Naby D.H., Khayyal M.T. (2011). The influence of irradiation on the potential chondroprotective effect of aqueous extract of propolis in rats. Int. J. Radiat. Biol..

[34] Zhang D., Huang B., Xiong C., Yue Z. (2015). Pinocembrin inhibits matrix metalloproteinase expression in chondrocytes. IUBMB Life.

[35] Freitas A.S., Cunha A., Cardoso S.M., Oliveira R., Almeida-Aguiar C. (2019). Constancy of the bioactivities of propolis samples collected on the same apiary over four years. Food Res. Int..

[36] Peixoto M., Freitas A.S., Cunha A., Oliveira R., Almeida-Aguiar C. (2021). Antioxidant and antimicrobial activity of blends of propolis samples collected in different years. Lwt.

[37] Peixoto M., Freitas A.S., Cunha A., Oliveira R., Almeida-Aguiar C. (2022). Mixing Propolis from Different Apiaries and Harvesting Years: Towards Propolis Standardization?. Antibiotics.

[38] Freitas A.S., Cunha A., Oliveira R., Almeida-Aguiar C. (2022). Propolis antibacterial and antioxidant synergisms with gentamicin and honey. J. Appl. Microbiol..

[39] Pereira L., Cunha A., Almeida-Aguiar C. (2022). Portuguese propolis from Caramulo as a biocontrol agent of the apple blue mold. Food Control.

[40] Moreira L., Dias L.G., Pereira J.A., Estevinho L. (2008). Antioxidant properties, total phenols and pollen analysis of propolis samples from Portugal. Food Chem. Toxicol..

[41] Miguel M.G., Nunes S., Dandlen S.A., Cavaco A.M., Antunes M.D. (2010). Phenols and antioxidant activity of hydro-alcoholic extracts of propolis from Algarve, South of Portugal. Food Chem. Toxicol..

[42] Valente M.J., Baltazar A.F., Henrique R., Estevinho L., Carvalho M. (2011). Biological activities of Portuguese propolis: Protection against free radical-induced erythrocyte damage and inhibition of human renal cancer cell growth in vitro. Food Chem. Toxicol..

[43] Cruz M., Antunes P., Paulo L., Ferreira A.M., Cunha A., Almeida-Aguiar C., Oliveira R. (2016). Antioxidant and dual dose-dependent antigenotoxic and genotoxic properties of an ethanol extract of propolis. RSC Adv..

[44] Valença I., Morais-Santos F., Miranda-Gonçalves V., Ferreira A.M., Almeida-Aguiar C., Baltazar F. (2013). Portuguese propolis disturbs glycolytic metabolism of human colorectal cancer in vitro. BMC Complement. Altern. Med..

[45] Silva-Carvalho R., Miranda-Gonçalves V., Ferreira A.M., Cardoso S.M., Sobral A.J.F.N., Almeida-Aguiar C., Baltazar F. (2014). Antitumoural and antiangiogenic activity of Portuguese propolis in in vitro and in vivo models. J. Funct. Foods.

[46] Oliveira R.D., Celeiro P., Barbosa-matos C., Freitas A.S., Cardoso S.M., Viana-pereira M., Almeida-aguiar C., Baltazar F. (2022). Portuguese Propolis Antitumoral Activity in Melanoma Involves ROS Production and Induction of Apoptosis. Molecules.

[47] Ahangari Z., Naseri M., Vatandoost F. (2018). Propolis: Chemical composition and its applications in endodontics. Iran. Endod. J..

[48] Silva J.C., Rodrigues S., Feás X., Estevinho L.M. (2012). Antimicrobial activity, phenolic profile and role in the inflammation of propolis. Food Chem. Toxicol..

[49] Singleton V.L., Orthofer R., Lamuela-Raventós R.M. (1999). [14] Analysis of total phenols and other oxidation substrates and antioxidants by means of folin-ciocalteu reagent. Scientia Horticulturae.

[50] Woisky R.G., Salatino A. (1998). Analysis of propolis: Some parameters and procedures for chemical quality control. J. Apic. Res..

[51] Tiveron A.P., Rosalen P.L., Franchin M., Lacerda R.C.C., Bueno-Silva B., Benso B., Denny C., Ikegaki M., De Alencar S.M. (2016). Chemical characterization and antioxidant, antimicrobial, and anti-inflammatory activities of South Brazilian organic propolis. PLoS ONE.

[52] Mitra K., Uddin N. (2014). Total Phenolics, Flavonoids, Proanthrocyanidins, Ascorbic Acid Contents and In-Vitro Antioxidant Activities of Newly Developed Isolated Soya Protein. Discourse J. Agric. Food Sci. JAFS.

[53] Kakkar P., Das B., Viswanathan P.N. (1984). A modified spectrophotometric assay of superoxide dismutase. Indian J. Biochem. Biophys..

[54] Zhang J., Cao X., Ping S., Wang K., Shi J., Zhang C., Zheng H., Hu F. (2015). Comparisons of ethanol extracts of Chinese propolis (poplar type) and poplar gums based on the antioxidant activities and molecular mechanism. Evidence-based Complement. Altern. Med..

[55] Mizushima Y., Kobayashi M. (1968). Interaction of anti-inflammatory drugs with serum proteins, especially with some biologically active proteins. J. Pharm. Pharmacol..

[56] Saso L., Valentini G., Casini M.L., Mattei E., Braghiroli L., Mazzanti G., Panzironi C., Grippa E., Silvestrini B. (1999). Inhibition of protein denaturation by fatty acids, bile salts and other natural substances: A new hypothesis for the mechanism of action of fish oil in rheumatic diseases. Jpn. J. Pharmacol..

[57] Neugebauer V., Han J.S., Adwanikar H., Fu Y., Ji G. (2007). Techniques for assessing knee joint pain in arthritis. Mol. Pain.

[58] Amorim D., David-Pereira A., Pertovaara A., Almeida A., Pinto-Ribeiro F. (2014). Amitriptyline reverses hyperalgesia and improves associated mood-like disorders in a model of experimental monoarthritis. Behav. Brain Res..

[59] Park E.H., Kahng J.H. (1999). Suppressive effects of propolis in rat adjuvant arthritis. Arch. Pharm. Res..

[60] Livy D.J., Parnell S.E., West J.R. (2003). Blood ethanol concentration profiles: A comparison between rats and mice. Alcohol.

[61] Mohammadzadeh S., Shariatpanahi M., Hamedi M., Ahmadkhaniha R., Samadi N., Ostad S.N. (2007). Chemical composition, oral toxicity and antimicrobial activity of Iranian propolis. Food Chem..

[62] Barton N.J., Strickland I.T., Bond S.M., Brash H.M., Bate S.T., Wilson A.W., Chessell I.P., Reeve A.J., McQueen D.S. (2007). Pressure application measurement (PAM): A novel behavioural technique for measuring hypersensitivity in a rat model of joint pain. J. Neurosci. Methods.

[63] Auh Q.-S., Ro J.Y. (2012). Effects of peripheral κ opioid receptor activation on inflammatory mechanical hyperalgesia in male and female rats. Neurosci. Lett..

[64] Hamers F.P., Lankhorst A.J., van Laar T.J., Veldhuis W.B., Gispen W.H. (2001). Automated quantitative gait analysis during overground locomotion in the rat: Its application to spinal cord contusion and transection injuries. J. Neurotrauma.

[65] Lankhorst A.J., ter Laak M.P., van Laar T.J., van Meeteren N.L.U., de Groot J.C.M.J., Schrama L.H., Hamers F.P.T., Gispen W.-H. (2001). Effects of Enriched Housing on Functional Recovery After Spinal Cord Contusive Injury in the Adult Rat. J. Neurotrauma.

[66] Gabriel A.F., Marcus M.A.E., Honig W.M.M., Walenkamp G.H.I.M., Joosten E.A.J. (2007). The CatWalk method: A detailed analysis of behavioral changes after acute inflammatory pain in the rat. J. Neurosci. Methods.

[67] Hall C.S. (1934). Emotional behavior in the rat. I. Defecation and urination as measures of individual differences in emotionality. J. Comp. Psychol..

[68] Prut L., Belzung C. (2003). The open field as a paradigm to measure the effects of drugs on anxiety-like behaviors: A review. Eur. J. Pharmacol..

[69] Nagai T., Sakai M., Inoue R., Inoue H., Suzuki N. (2001). Antioxidative activities of some commercially honeys, royal jelly, and propolis. Food Chem..

[70] Leelaprakash G., Mohan Dass S. (2011). Invitro anti-inflammatory activity of methanol extract of enicostemma axillare. Int. J. Drug Dev. Res..

[71] Bhaskar V.H., Mohite P.B. (2010). Design, Synthesis, Characterization and Biological Evaluation of Some Novel 1, 5 Disubstituted Tetrazole As Potential Anti-Inflammatory Agents. J. Optoelectron. Biomed. Mater..

[72] Govindappa M., Naga Sravya S., Poojashri M.N., Sadananda T.S., Chandrappa C.P., Santoyo G., Sharanappa P., Anil Kumar N.V. (2011). Antimicrobial, antioxidant and in vitro anti-inflammatory activity and phytochemical screening of water extract of *Wedelia trilobata* (L.) hitchc. J. Med. Plant Res..

[73] Muramatsu Y., Sasho T., Saito M., Yamaguchi S., Akagi R., Mukoyama S., Akatsu Y., Katsuragi J., Fukawa T., Endo J. (2014). Preventive effects of hyaluronan from deterioration of gait parameters in surgically induced mice osteoarthritic knee model. Osteoarthr. Cartil..

[74] Bailey K.R., Crawley J.N. (2009). Anxiety-Related Behaviors in Mice. Methods of Behavior Analysis in Neuroscience.

[75] Kara H., Çağlar C., Asiltürk M., Karahan S., Uğurlu M. (2021). Comparison of a manual walking platform and the CatWalk gait analysis system in a rat osteoarthritis model. Adv. Clin. Exp. Med..

[76] Walsh R.N., Cummins R.A. (1976). The open-field test: A critical review. Psychol. Bull..

[77] Miguel M.G. (2013). Chemical and biological properties of propolis from the western countries of the Mediterranean basin and Portugal. Int. J. Pharm. Pharm. Sci..

[78] Falcão S.I., Vale N., Gomes P., Domingues M.R.M., Freire C., Cardoso S.M., Vilas-Boas M. (2013). Phenolic profiling of Portuguese propolis by LC-MS spectrometry: Uncommon propolis rich in flavonoid glycosides. Phytochem. Anal..

[79] Barlak Y., Deǧer O., Çolak M., Karatayli S.C., Bozdayi A.M., Yücesan F. (2011). Effect of Turkish propolis extracts on proteome of prostate cancer cell line. Proteome Sci..

[80] Falcão S.I., Vilas-Boas M., Estevinho L.M., Barros C., Domingues M.R.M., Cardoso S.M. (2010). Phenolic characterization of Northeast Portuguese propolis: Usual and unusual compounds. Anal. Bioanal. Chem..

[81] Oroian M., Ursachi F., Dranca F. (2020). Influence of ultrasonic amplitude, temperature, time and solvent concentration on bioactive compounds extraction from propolis. Ultrason. Sonochem..

[82] Kubiliene L., Laugaliene V., Pavilonis A., Maruska A., Majiene D., Barcauskaite K., Kubilius R., Kasparaviciene G., Savickas A. (2015). Alternative preparation of propolis extracts: Comparison of their composition and biological activities. BMC Complement. Altern. Med..

[83] Basyirah N., Muslim M., Rodi M., Mohd K., Zin M., Azemin A., Mohd S. (2018). Chemical Composition and Antioxidant Activity of Stingless Bee Propolis from Different Extraction Methods. Int. J. Eng. Technol..

[84] Mursu J., Voutilainen S., Nurmi T., Tuomainen T.-P., Kurl S., Salonen J.T. (2008). Flavonoid intake and the risk of ischaemic stroke and CVD mortality in middle-aged Finnish men: The Kuopio Ischaemic Heart Disease Risk Factor Study. Br. J. Nutr..

[85] Sheng J., Zhou J., Wang L., Xu J., Hu Q. (2007). Antioxidant activity of ethanol and petroleum ether extracts from Brazilian propolis. Eur. Food Res. Technol..

[86] Brand-Williams W., Cuvelier M.E., Berset C. (1995). Use of a free radical method to evaluate antioxidant activity. LWT-Food Sci. Technol..

[87] Hazra B., Biswas S., Mandal N. (2008). Antioxidant and free radical scavenging activity of Spondias pinnata. BMC Complement. Altern. Med..

[88] Badarinath A.V., Rao K.M., Madhu C., Chetty S., Ramkanth S., Rajan T.V.S., Gnanaprakash K. (2010). A Review On In-Vitro Antioxidant Methods: Comparisions, Correlations and Considerations. Int. J. PharmTech Res..

[89] Cunha I.B.S., Sawaya A.C.H.F., Caetano F.M., Shimizu M.T., Marcucci M.C., Drezza F.T., Povia G.S., Carvalho P.d.O. (2004). Factors that influence the yield and composition of Brazilian propolis extracts. J. Braz. Chem. Soc..

[90] Irigoiti Y., Navarro A., Yamul D., Libonatti C., Tabera A., Basualdo M. (2021). The use of propolis as a functional food ingredient: A review. Trends Food Sci. Technol..

[91] Moret S., Purcaro G., Conte L.S. (2010). Polycyclic aromatic hydrocarbons (PAHs) levels in propolis and propolis-based dietary supplements from the Italian market. Food Chem..

[92] Kowalski S., Makarewicz M. (2017). Functional properties of honey supplemented with bee bread and propolis. Nat. Prod. Res..

[93] Sakat S.S., Juvekar A.R., Gambhire M.N. (2010). In-vitro antioxidant and anti-inflammatory activity of methanol extract of Oxalis corniculata linn. Int. J. Pharm. Pharm. Sci..

[94] Rayiti R., Munnangi S., Bandarupalli R., Chakka V., Nimmagadda S., Sk L., Uppalapati S., Bolla R., Challa S. (2020). Effect of chrysin on mechanical hyperalgesia in chronic constriction injury-induced neuropathic pain in rat model. Int. J. Appl. Basic Med. Res..

[95] Rao B.G., Rao P.U., Rao E.S., Rao T.M., Praneeth. D V.S. (2012). Evaluation of in-vitro antibacterial activity and anti-inflammatory activity for different extracts of Rauvolfia tetraphylla L. root bark. Asian Pac. J. Trop. Biomed..

[96] Hu F., Hepburn H.R., Li Y., Chen M., Radloff S.E., Daya S. (2005). Effects of ethanol and water extracts of propolis (bee glue) on acute inflammatory animal models. J. Ethnopharmacol..

[97] Teeple E., Jay G.D., Elsaid K.A., Fleming B.C. (2013). Animal Models of Osteoarthritis: Challenges of Model Selection and Analysis. AAPS J..

[98] Ji G., Fu Y., Ruppert K.A., Neugebauer V. (2007). Pain-Related Anxiety-Like Behavior Requires CRF1 Receptors in the Amygdala. Mol. Pain.

