# Supplementary material for: An Insight on the Biomedical Potential of Portuguese Propolis from Gerês
Source: Foods. 2022 Oct 29;11(21):3431. doi: 10.3390/foods11213431 (PMC9656172; doi:10.3390/foods11213431)
Supplement: Supplementary file 1 [file foods-11-03431-s001.zip › foods-1972365-supplementary.pdf]

foods MDPI

Supplementary Material

Supplementary Table S1 – Total phenolic content (TPC) and total flavonoid content (TFC) of the hydroalcoholic extracts of propolis from Gerês (G.EEs)and the resultant mixtures (mG). Results are expressed as mean ± SD. (G: Gerês; mG: a mixture of propolis har- vested over 5 years; EE: ethanol extract; mg GAE/g: milligram of gallic acids equivalents per gram of EE; mg QE/g: milligram of quercetin equivalents per gram of EE).

	**Total Phenolic Content (TPC)** (mg GAE/g)		**Total Flavonoid Content (TFC)** (mg QE/g)	
	**Ethanol 70% (*v*/*v*)**	**Ethanol 35% (*v*/*v*)**	**Ethanol 70% (*v*/*v*)**	**Ethanol 35% (*v*/*v*)**
**G11.EE**	71.00 ± 13.31	72.08 ± 12.09	41.86 ± 4.29	30.49 ± 3.85
**G12.EE**	103.34 ± 18.58	92.21 ± 9.90	46.16 ± 3.99	34.11 ± 3.37
**G13.EE**	81.34 ± 14.48	67.99 ± 6.99	40.73 ± 5.25	33.99 ± 5.26
**G14.EE**	74.58 ± 11.20	67.61 ± 7.68	44.30 ± 4.88	38.43 ± 5.72
**G15.EE**	112.86 ± 19.24	101.36 ± 10.81	57.55 ± 6.69	34.31 ± 3.31
**mG.EE**	96.13 ± 18.35	89.97 ± 14.84	56.67 ± 4.65	35.76 ± 4.71

Supplementary Table S2 - DPPH• scavenging activity of hydroalcoholic extracts of propolis from Gerês. Results are expressed in EC_{50} (μg/mL) as mean ± standard deviation (SD). (DPPH: 2,2-diphenyl-2-picry-lhydrazyl; G: Gerês; mG: a mixture of propolis samples).

	**DPPH• Scavenging Activity** EC50 (μg/mL)	
	**Ethanol 70% (*v*/*v*)**	**Ethanol 35% (*v*/*v*)**
**G11.EE**	9.87 ± 0.32	18.54 ± 0.87
**G12.EE**	4.71 ± 0.76	12.40 ± 0.18
**G13.EE**	16.69 ± 1.57	19.16 ± 0.53
**G14.EE**	7.39 ± 0.40	19.05 ± 1.05
**G15.EE**	5.42 ± 0.59	12.19 ± 1.05
**mG.EE**	4.73 ± 0.36	17.01 ± 0.61

Supplementary Table S3 - Superoxide anion scavenging activity of hydroalcoholic extracts of propolis from Gerês**.** Results are expressed in EC_{50} (μg/mL) as mean ± standard deviation (SD). (G: Gerês; mG: a mixture of propolis samples).

	**Superoxide Anion Scavenging Activity** EC50 (μg/mL)	
	**Ethanol 70% (*v*/*v*)**	**Ethanol 35% (*v*/*v*)**
**G11.EE**	136.00 ± 15.32	179.54 ± 19.61
**G12.EE**	59.71 ± 1.97	122.14 ± 7.16
**G13.EE**	162.11 ± 19.39	185.16 ± 23.86
**G14.EE**	191.87 ± 7.26	236.56 ± 5.45
**G15.EE**	107.66 ± 3.97	101.07 ± 4.82
**mG.EE**	118.11 ± 9.52	175.26 ± 21.56

Supplementary Table S4 - Inhibition of heat-induced BSA denaturation by hydroalcoholic extracts of Gerês propolis. Results were expressed as mean ± standard deviation (SD). (BSA: Bovine serum albumin; G: Gerês; mG: a mixture of propolis samples).

	**% Inhibition of BSA Denaturation**	
	**Ethanol 70% (*v*/*v*)**	**Ethanol 35% (*v*/*v*)**
**G11.EE**	61.66 ± 4.81	74.69 ± 5.27
**G12.EE**	24.93 ± 6.27	58.51 ± 2.49
**G13.EE**	53.15 ± 8.40	62.18 ± 3.74
**G14.EE**	52.91 ± 8.82	72.21 ± 5.96
**G15.EE**	60.17 ± 2.58	53.80 ± 7.21
**mG.EE**	65.55 ± 2.55	60.87 ± 2.94
